# Practical Notes on Diagnosis and Treatment

**Published:** 1908-08-22

**Authors:** 


### Practical Notes on Diagnosis and Treatment.

**Fœtid Bronchitis.**

In this the following inhalation may be ordered:—Carbolic acid ʒss., tinc. camph. co ℥iij. A teaspoonful to be inhaled from half a pint of hot water.

**Chronic Constipation.**

In many cases of simple constipation the injection of a drachm or two of olive oil into the rectum at bed time will produce a satisfactory evacuation the following morning.—*Dr. Knowsley Sibley.*

**Venesection in Uræmia.**

When an attack of uræmia comes on suddenly in acute nephritis in a previously robust patient in whom the blood-pressure is high, 12 to 24 ounces of blood may be drawn from a vein with great advantage.

**Leucoderma.**

Leucoderma may occur at any age, though the middle period of life is that at which it is most common. It has been observed as early as the fourth year, and it has also been known to commence in advanced old age.—*Mr. Wilmott H. Evans.*

**Laryngismus.**

Few cases of laryngismus resist treatment by suitable diet and by castor oil, suspended in mucilage, with rhubarb and bromide of potassium; belladonna or chloral hydrate may be added, especially if convulsions have occurred.—*Dr. Leonard Guthrie.*

**The Gouty Inheritance.**

However difficult it may be to understand, observations make it certain that the offspring of gouty parents are specially liable to the painful neuroses, and this is particularly true of those who are of the female sex. Thus, in the more marked forms of migraine, parental or ancestral gout is seldom absent.—*Sir Wm. Gowers.*

**Diabetes Insipidus.**

Some cases of this disease are reported as showing great improvement under strychnine given by hypodermic injection.

**Morphinomania.**

Dr. Mary S. P. Strangman reports a case of morphinomania of 30 years' standing treated successfully by the use of atropine and strychnine.

**Tobacco Amblyopia.**

When the symptoms in a case of tobacco amblyopia have not persisted for more than three months, recovery is usually complete, provided that absolute abstention from tobacco is practised for at least 12 months.

**Threatened Abortion.**

Absolute rest in the recumbent position is essential. If there is bleeding but no pain small doses of ergot may be given. If there are pains as well as bleeding it is better to give small doses of opium.—*Dr. W. J. Gow.*

**Standardisation of Digitalis.**

I unhesitatingly express the belief that many hundreds of patients die annually from digitalis and its allies not possessing the virtues which are required of them. The remedy is to standardise these drugs by physiological means.—*Professor W. E. Dixon.*

**Hæmoptysis.**

In the hæmoptysis of pulmonary phthisis the use of circulatory depressants is desirable. From this point of view ipecacuanha is most serviceable. It should be given in small doses, repeated every one or two hours. It is not necessary or desirable to produce emetic effects.—*Dr. R. W. Philip.*

